# The bizzare phenomenon of smokers’ paradox in the immediate outcome post acute myocardial infarction: an insight into the Malaysian National Cardiovascular Database-Acute Coronary Syndrome (NCVD-ACS) registry year 2006–2013

**DOI:** 10.1186/s40064-016-2188-3

**Published:** 2016-04-26

**Authors:** Padmaa Venkatason, Norsabihin Mohd Salleh, Yong Zubairi, Imran Hafidz, Wan Azman Wan Ahmad, Sim Kui Han, Ahmad Syadi Mahmood Zuhdi

**Affiliations:** Cardiology Unit, Department of Medicine, University Malaya Medical Centre, Kuala Lumpur, Malaysia; Center for Foundation Studies in Science, University of Malaya, Kuala Lumpur, Malaysia; National Heart Association of Malaysia, Jalan Tun Razak, Kuala Lumpur, Malaysia

## Abstract

**Background:**

‘Smoker’s paradox’ is a controversial phenomenon of an unexpected favourable outcome of smokers post acute myocardial infarction. There are conflicting evidences from the literature so far. We investigate for the existence of this phenomenon in our post acute myocardial infarction patients.

**Methods:**

We analysed 12,442 active smokers and 10,666 never-smokers diagnosed with STEMI and NSTEMI from the Malaysian National Cardiovascular Database-Acute Coronary Syndrome (NCVD-ACS) year 2006–2013 from 18 hospitals across Malaysia. Comparisons in the baseline characteristics, clinical presentation, in-hospital treatment and short term clinical outcome were made between the two groups. To compare the clinical outcome, an extensive multivariate adjustment was made to estimate the allcause mortality risk ratios for both groups.

**Results:**

The active smokers were younger (smokers 53.7 years vs non-smokers 62.3 years *P* < 0.001) and had lower cardiovascular risk burden and other co-morbidities. STEMI is more common in smokers and intravenous thrombolysis was the main reperfusion therapy in both groups. Smokers had a higher rate of in-hsopital coronary revascularisation in NSTEMI group (21.6 % smokers vs 16.7 % non-smokers *P* < 0.001) but similar to non-smokers in the STEMI group. Multivariate adjusted mortality risk ratios showed significantly lower mortality risks of smokers at both in-hospital (RR 0.510 [95 % CI 0.442–0.613]) and 30-day post discharge (RR 0.534 [95 % CI 0.437–0.621]).

**Conclusion:**

Smoking seems to be associated with a favourable outcome post myocardial infarction. The phenomenon of ‘smoker’s paradox’ is in fact a reality in our patients population. The definitive explanation for this unexpected protective effect of smoking remains unclear.

## Background

Smoking is a well established risk factor for cardiovascular disease (Chen and Boreham 2002). ‘Smoker’s paradox’ is, however, an observational phenomenon of an unexpected favourable outcome in smokers post acute myocardial infarction (MI). There has been great interest in this controversy over the past decades. Some suggest that the paradoxical favourable outcome is due to the more ‘thrombotic’ nature of MI in smokers as oppose to atherosclerotic in non-smokers and hence better reperfusion response after thrombolyis (Grines et al. 1995; Zahger et al. 1995). Others argue that smokers were younger at the onset with better baseline prognostic factors such as lower rate of diabetes and hypertension among others (Weisz et al. 2005; Kelly et al. 1985). Nevertheless there is no universally accepted satisfactory explanation for this. Thus, we investigate for the existence of ‘smoker’s paradox’ in our post-MI population by means of anonymous patients data from the Malaysian National Cradiovascular Database registry-Acute coronary syndrome (NCVD-ACS) year 2006–2013.

## Methods

### Study subjects and data collection

The Malaysian National Cardiovascular Disease Database-Acute Coronary Syndrome (NCVD-ACS) registry was used to identify patients from 17 hospitals across Malaysia admitted with ST-elevation MI (STEMI) and non-ST elevation MI (NSTEMI) from the year 2006 to 2013. NVCD registry is a prospective registry sponsored by the Ministry of Health, Malaysia (MoH) and co-sponsored by the National Heart Association of Malaysia (NHAM). A total of 23,108 patients were identified for this study. Patients selected were either active smokers (n = 12,442) or non-smokers (n = 10,666). Active smokers were defined as patients having regularly smoked tobacco products/product (includes cigarettes, cigars or pipes) one or more times per day or have smoked within the 30 days prior to the admission. Non-smokers were defined as patients who have never smoked for their entire lives. Baseline characteristics, clinical presentation, in-hospital treatment, procedural details and clinical outcome at discharge and at 30 days post discharge. Record matching against the National Death Register, Malaysia was performed to verify the mortality status (alive or dead) of all study patients.

### Statistical analysis

Continuous variables were expressed as mean ± standard deviation and median (IQR) if skewed. The differences between active smokers and non-smokers were analysed using *t* test or Wicoxon rank sum test (if skewed). Categorical variables were described as numbers (percentages), and differences were analysed using the Chi-square test or the Fisher exact test. To hinder biases in the estimates and loss of power, missing data for explanatory variables were assumed to be missing at random. A generalized linear model with a log link, binomial distribution, and a robust variance estimator was used to estimate the risk ratios. The risk ratios represent the relative risk for mortality of the patients in non-smokers compared to active smokers. Variables that were statistically significantly different (a 2-sided *P* value of less than 0.05) between active smokers and non-smokers, that were of clinical importance, and that had sufficient outcomes in the respective subcategories were adjusted for in the model. Statistical significance was considered if the *P* value was less than 0.05 and the 95 % confidence intervals of risk ratios excluded the value of 1. All analyses were conducted using SPSS statistical software (version 21, IBM SPSS Statistics, USA).

### Ethics approval and consent

This NCVD registry study was approved by the Medical Review & Ethics Committee (MREC), MOH Malaysia in 2007 (Approval Code: NMRR-07-20-250). MREC waived informed consent for NCVD.

## Results

### Patients baseline characteristics (Table 1)

#### Demographics (Table 1)

Active smokers were significantly younger (53.7 vs 62.3 years *P* < 0.001) with higher male to female dominancy (97.9 vs 53.6 % *P* < 0.001) and Malay racial group (59.9 vs 45.7 % *P* < 0.001).

**Table 1:** Patients’ baseline characteristics

	Active smokers (n = 12,442)	Non-smokers (n = 10,666)	*P* value
Age			
Mean	53.7 (53.46, 53.83)	62.3 (62.09, 62.45)	<0.001*
Standard deviation	11.29	11.64	
Gender			
Male	12182 (97.9 %)	5712 (53.6 %)	<0.001
Female	260 (2.1 %)	4954 (46.4 %)	
Ethnicity			
Malay	7398 (59.5 %)	4879 (45.7 %)	
Chinese	2173 (17.5 %)	2409 (22.6 %)	<0.001
Indian	1920 (15.4 %)	2755 (25.8 %)	
Others	951 (7.6 %)	623 (5.8 %)	
Risk factors/co-morbidities			
Dyslipidaemia	2933 (23.6 %)	3838 (36.0 %)	<0.001
Hypertension	5267 (42.3 %)	7390 (69.3 %)	<0.001
Diabetes	3691 (29.7 %)	5627 (52.8 %)	<0.001
Family history premature CAD	1595 (12.8 %)	1051 (9.9 %)	<0.001
Cerebrovascular disease	228 (1.8 %)	469 (4.4 %)	<0.001
Peripheral vascular disease	43 (0.3 %)	97 (0.9 %)	<0.001
Chronic lung disease	276 (2.2 %)	264 (2.5 %)	<0.001
Congestive heart failure	416 (3.3 %)	885 (8.3 %)	<0.001
Chronic renal failure	311 (2.5 %)	1028 (9.6 %)	<0.001
ACS diagnosis			
STEMI	9609 (77.2 %)	5684 (53.3 %)	<0.001
NSTEMI	2833 (22.8 %)	4982 (46.7 %)	
Killip class			
Killip 1	6712 (66.1 %)	5092 (61.9 %)	
Killip 2	2081 (20.5 %)	1951 (23.7 %)	<0.001
Killip 3	429 (4.2 %)	515 (6.3 %)	
Killip 4	939 (9.2 %)	672 (8.2 %)	

*STEMI* ST elevation myocardial infarction, *NSTEMI* non-ST elevation myocardial infarction

All *P* values are calculated using the Chi-square test unless stated

* *T* test

#### Cardiovascular risk factors and co-morbidities (Table 1)

Active smokers showed a better cardiovascular risk factor profile with significantly lower rate of diabetes mellitus (29.7 vs 52.8 % *P* < 0.001), hypertension (42.3 vs 69.3 % *P* < 0.001) and dyslipidaemia (23.6 vs 36.0 % *P* < 0.001). However the active smokers have higher rate of family history of premature coronary artery disease (CAD) (12.8 vs 9.9 % *P* < 0.001).

Other co-morbidities of previous cerebrovascular disease, congestive cardiac failure, chronic renal failure, chronic lung disease and peripheral vascular disease were also significantly lower in the active smoking group.

#### ACS presentation and killip class (Table 1)

There is a significantly higher rate of STEMI in active smokers compared to the non-smokers (77.2 vs 53.3 % *P* < 0.001). Within STEMI patients, the smokers had more patients who presented without clinical evidence of LV dysfunction (Killip 1) (66.1 vs 61.9 % *P* < 0.001).

### In-hospital acute treatment, coronary revascularisation and evedence-based pharmacotherapy (Table 2)

#### Acute reperfusion therapy (Table 2)

Majority of the STEMI patients received IV thrombolysis (smokers 77.1 % vs non-smokers 69.0 %) with only a small minority had primary PCI done (smokers 9.5 % vs non-smokers 11.3 %). The median door to needle time was significantly shorter in the smokers’ group (smokers 45 min vs non smokers 50 min).

**Table 2:** In-hospital acute treatment, coronary revascularisation and evidence-based pharmacotherapy

	Active smokers	Non-smokers	*P* value
Thrombolysis			
Given	7239 (77.1 %)	3826 (69.0 %)	<0.001
Not given—proceeded to primary angioplasty	891 (9.5 %)	627 (11.3 %)	
Not given—missed	961 (10.2 %)	807 (14.6 %)	
Not given—patient refusal	34 (0.4 %)	20 (0.4 %)	
Not given—contraindicated	269 (2.9 %)	252 (4.5 %)	
Door to needle time (median)	45 (26–82)	50 (30–94)	<0.001*
STEMI			
Percutaneous coronary intervention	2679 (29.8 %)	1541 (29.2 %)	<0.001
NSTEMI			
Percutaneous coronary intervention	566 (21.6 %)	773 (16.7 %)	<0.001
Evidence-based Medications			
Aspirin	10,445 (93.8 %)	8370 (87.9 %)	<0.001
ADP-antagonist	6629 (78.4 %)	5462 (73.0 %)	<0.001
ACE-I/ARB	6575 (53.4 %)	5422 (52.3 %)	0.080
Beta blocker	7301 (68.4 %)	6350 (68.2 %)	0.761
Statin	10,024 (90.5 %)	8179 (86.3 %)	<0.001

*ACE-I* angiotensin converting enzyme inhibitor, *ARB* angiotensin receptor blocker, *ADP* adenosine diphosphate

All *P* values are calculated using the Chi-square test unless stated

* Wicoxon rank sum test

#### In-hospital coronary angiogram/angioplasty and pharmacotherapy (Table 2)

In the STEMI group, the smokers received similar rate of invasive coronary revascularisation (29.8 % smokers vs 29.2 % non-smokers). However in NSTEMI, the invasive revascularisatio rate is significantly higher in smokers (21.6 % smokers vs 16.7 % non-smokers). In terms of the evidence-based pharmacotherapy, more active smokers received aspirin (93.8 % smokers vs 87.9 % non-smokers) and statin (90.5 % smokers vs 86.3 % non-smokers) compared to the non-smokers. The rate of other medications were the same for both groups.

### Clinical outcome (all-cause mortality) (Tables 3 and 4)

#### In-hospital all-cause mortality (Table 3)

The in-hospital mortality rates for STEMI, NSTEMI and overall groups were all consistently lower in active smokers compared to the non smokers. Despite extensive multi-variate adjustments of all confounding factors, the mortality risk ratios remained significantly lower in smokers across all groups. STEMI RR 0.628 [95 % CI 0.586–0.673] *P* < 0.001, NSTEMI RR 0.423 [95 % CI 0.369–0.575] *P* value <0.001, and overall RR 0.510 [95 % CI 0.442–0.613] *P* < 0.001.

**Table 3:** Non-adjusted and adjusted in-hospital mortality risk ratios of active smokers and non-smokers

ACS type	Number of patients	Deaths, n (%)	Unadjusted risk ratio (95 % CI)	*P* value	Adjusted risk ratio (95 % CI)	*P* value
All (n = 23,108)						
Active smokers	12,442	699 (5.6 %)	0.330 (0.311, 0.350)	<0.001	0.510 (0.442, 0.613)*	<0.001
Non-smokers	10,666	1026 (9.6 %)	1		1	
STEMI (n = 15,293)						
Active smokers	9609	566 (5.9 %)	0.358 (0.293, 0.436)	<0.001	0.628 (0.586, 0.673)*	<0.001
Non smokers	5684	645 (11.3 %)	1		1	
NSTEMI (n = 7815)						
Active smokers	2833	133 (4.7 %)	0.328 (0.309, 0.348)	<0.001	0.423 (0.369, 0.575)*	<0.001
Non smokers	4982	381 (7.6 %)	1		1	

* Risk ratios were adjusted with respect to each variable with significant *P* value (<0.05) in the univariate analysis (refer Tables 1, 2)

**Table 4:** Non-adjusted and adjusted 30-day mortality risk ratios of active smokers and non-smokers

ACS type	Number of patients	Deaths, n (%)	Unadjusted risk ratio (95 % CI)	*P* value	Adjusted risk ratio (95 % CI)	*P* value
All (n = 23,108)						
Active smokers	12,442	892 (7.2 %)	0.338 (0.322, 0.362)	<0.001	0.534 (0.437, 0.621)*	<0.001
Non-smokers	10,666	1317 (12.3 %)	1		1	
STEMI (n = 15,293)						
Active smokers	9609	714 (7.4 %)	0.398 (0.317, 0.499)	<0.001	0.635 (0.592, 0.681)*	<0.001
Non smokers	5684	776 (13.7 %)	1		1	
NSTEMI (n = 7815)						
Active smokers	2833	178 (6.3 %)	0.336 (0.316, 0.356)	<0.001	0.489 (0.421, 0.589)*	<0.001
Non smokers	4982	541 (10.9 %)	1		1	

* Risk ratios were adjusted with respect to each variable with significant *P* value (<0.05) in the univariate analysis (refer Tables 1, 2)

#### 30-day post discharge all-cause mortality (Table 4)

The mortality rate and the unadjusted mortality risk ratios remained lower in the active smokers at 30-day post discharge within all groups (STEMI, NSTEMI and overall). Again, even after extensive confounding multivariate risk adjustments, the risk ratios for active smokers were found to be significantly lower than non-smokers across all groups. STEMI RR 0.635 [95 % CI 0.592–0.681] *P* value <0.001, NSTEMI RR 0.489 [95 % CI 0.421–0.589] *P* value <0.001 and Overall RR 0.534 [95 % CI 0.437–0.621] *P* value <0.001.

## Discussion

Our population has high prevalence of smoking. According to the National Health and Morbidity Survey (NHMS) Malaysia, the prevalence of adult male smokers in Malaysian population is 46.5 %, highest among the Malays (55.9 %), lower education group and rural areas (Lim et al. 2013). Reports from other regions regarding the unexpected favourable outcome of smokers post myocardial infarction has dated back to the pre-thrombolytic era of STEMI (Kelly et al. 1985; Helmers 1973). There have been great debates in the literature on this controversial topic and it has oftenly been disputed mainly on the confounding age and pre-morbid differences which favour smokers than the non-smokers in general (Grines et al. 1995; Barbash et al. 1995; Zuhdi et al. 2013).

Nevertheless, the main pathogenesis of acute myocardial infarction in smokers has been reported to differ from those of non-smokers. There is documented increase in haematocrit and fribrinogen levels in smokers which predispose them more to intra-coronary thrombosis (Sambola et al. 2003). It is thus suggested that the coronary flow limitation is largely due to thrombogenic obstruction with minimal underlying atherosclerotic narrowing. Where as, in non-smokers it is the atherosclerotic narrowing which is predominant (Grines et al. 1995; Zahger et al. 1995). With this came the theory of fibrin-rich thrombus in smokers which is said to be more susceptible to thrombolysis/anti-coagulation and theoretically leads to the seemingly better outcome of smokers particularly in the thrombolytic era (Aune et al. 2011). Some other hypotheses include higher case-fatality among smokers even before reaching the hospitals (Sonke et al. 1997) and the ischaemic preconditioning effect of tobacco (Gupta et al. 2014). However, these remain as theories without sufficient data for definitive proof to relate them to the favourable clinical outcome of smokers.

In one study, the response in myocardial reperfusion after thrombolytic therapy in smokers were objectively assessed and compared to non-smokers in a study using TIMI myocardial perfusion grade (TMPG) which showed better response in smokers (Kirtane et al. 2005). However more recent data in the era of primary PCI showed no significant difference betweeen smokers and non-smokers (De Luca et al. 2014; Allahwala et al. 2013) and smokers benefit equally from PCI and thrombolysis (Rasmussen et al. 2012).

Our study was done in the background of IV thrombolysis as the dominant reperfusion strategy for STEMI. Only a small minority (<10 %) of STEMIs underwent primary PCI and overall in-hospital coronary revascularisation was around 30 %. The NSTEMI patients were treated mainly with non-invasive pharmacotherapy with only around 20 % received in hospital coronary revascularisation. After extensive covariates adjustments, we observed that active smokers had a (overall) significantly lower in-hospital mortality risk compared to those who never smoked. Sub-group analysis of STEMI and NSTEMI were both consistent with a lower adjusted mortality risk compared to non-smokers. At 30-day post discharge, the trend in lower adjusted mortality risks were sustained across all groups with smokers maintaining the protective effect.

However this study only looked at the immediate clinical outcome (in-hospital and 30-day post discharge). Patients’ smoking status after the myocardial infarction was not assessed and hence the sustainability of the favourable effect of smoking in the long run for our patients remains unknown. Some recent data showed that smoking is actually related to increased long term mortality (Robertson et al. 2014; Takagi et al. 2014) and quitting smoking is associated with improved survival after acute coronary syndromes (Gerber et al. 2009; Chow et al. 2010). This comes as the suggestion that cigarette smoking may increase platelet aggregation in patients with ischemic heart disease in an aspirin nonresponsive manner (Pamukcu et al. 2011). All these data suggests that favourable smokers’ paradox effect is only limitted to short term outcome if it does exist at all.

The observed phenomenon of smokers’ paradox is thus a highly controversial one. Many other studies have refuted the existence of this phenomenon (Andrikopoulos et al. 2001; Gottlieb et al. 1996). A study showed that smokers had better early outcome but the benefit rapidly vanishes in the late phase (Takagi et al. 2014) and in the event of post coronary revascularisation (PCI or CABG), smoking is associated with adverse outcome (Zhang et al. 2015; Yi-Hwei et al. 2013).

Collectively, current data suggests that even though smoking may be associated with a favourable early outcome, the overall picture is still not in favour of smoking especially on long term outcome and post coronary revascularisation. This study did not assess the smoking status post myocardial infarction and based on the above evidence from the literature, the favourable outcome of smokers are short-lived and hence we continue to strongly advocate the importance of smoking cessation.

## Conclusion

The phenomenon of ‘smoker’s paradox’ is in fact a reality among our patients and is unlikely due to just plain bias from the favourable baseline characteristics of smokers. Even after extensive covariates adjustment for all the favourable baseline characteristics in active smokers, the reduction in mortality risks persists. In conclusion, ‘smoker’s paradox’ in the immediate outcome post myocardial infarction was observed in our patients’ population. Active smokers tend to do better at both in-hospital and 30-days post discharge with significantly lower overall mortality risk compared to those patients who never smoked. The real explanation to this bizarre outcome however, remains unclear.

Nevertheless the study is limitted to only immediate outcome. Smoking status post moyocardial infarction and its effect on long term outcome was not assesed. Currently, the overall evidence from the literature is still in favour of non-smokers especially for long term outcome and post coronary revascularisations.
